# Longitudinal study of mental health changes in residents affected by an initial outbreak of COVID-19 in China

**DOI:** 10.3389/fpubh.2022.1019703

**Published:** 2023-01-09

**Authors:** Na Du, Yu Xiao, Yingjie Ouyang, Yunge Li, Ting Geng, Chunya Li, Chan Yu, Yalan Hu, Fengyu Liu, Li Zhang, Min Zhu, Lishi Luo, Juan Huang

**Affiliations:** ^1^Department of Clinical Psychology, The Fourth People's Hospital of Chengdu, Chengdu, Sichuan, China; ^2^Department of Respiratory Medicine, The Eighth People's Hospital of Chengdu, Chengdu, Sichuan, China

**Keywords:** anxiety, COVID-19, depression, mental health, follow-ups

## Abstract

**Introduction:**

The COVID-19 pandemic is ongoing, and the world continues to work to defeat it. We designed this study to understand the longitudinal change in the mental health of residents who experienced the initial disease outbreak in China and to explore the long-term influencing factors.

**Methods:**

The Perceived Stress Scale (PSS), Generalized Anxiety Scale (GAD-7), and Patient Health Questionnaire-9 (PHQ-9) were administered to the same sample four times: during the initial outbreak (T1), 1 month later (T2), 18 months later (T3), and 26 months later (T4).

**Results:**

A total of 397 participants completed all of the follow ups. The mean PSS scores among the four time points showed significant differences (*F* = 183.98, *P* < 0.001), with the highest score at T1 (15.35 ± 7.14), a sharp decline at T2 (11.27 ± 6.27), an obvious rebound at T3 (15.17 ± 7.46), and finally a slight decrease at T4 (14.41 ± 7.99). Among the four mean GAD-7 scores, significant differences were also found (*F* = 242.0, *P* < 0.001), with the trend that from T1 (7.42 ± 6.03) to T2 (7.35 ± 5.88), the scores remained steady, while they showed an apparent decline at T3 (5.00 ± 5.30) and no obvious change at T4 (4.91 ± 4.81). There were no significant differences among the mean PHQ-9 scores (*F* = 1.256, *P* < 0.284). The long-term influencing factors differed for stress, anxiety and depression, but all three were influenced by a history of psychosis at T4, quarantine status and whether the participants' family members were infected during the initial outbreak.

**Discussion:**

The survey revealed that repeated outbreaks in other areas also had an impact on those who experienced the initial outbreak, with a return of stress, a decline in anxiety, and no change in depression, which provides direction for interventions in the future.

## 1. Introduction

On January 23, 2020, Wuhan became the city first affected by the Hubei Province outbreak of COVID-19. One week after the unprecedented catastrophe, on January 30, the World Health Organization (WHO) declared the outbreak a public health emergency of international concern (1). At the initial stage, little was known about this new virus, and effective treatment was lacking. The infected patients usually suffered from severe respiratory symptoms, and the death rate was relatively high (2). Because of these factors, individuals experienced various mental health problems immediately after the initial outbreak, and the most common symptoms included acute stress reactions, anxiety, and depression (3–6), which also became the main focus of research on the impact of COVID-19 on mental health.

However, with the continual evolution of the virus and the development of vaccines, the symptoms of infection eased and became mild or even asymptomatic (7). Many Western countries gradually lifted the COVID-19 precautions that were in place to prevent the spread of the pandemic, such as social distancing, mask wearing, public and private gatherings, and reopened schools (8–10). However, in contrast to Western countries, China continued to enforce strict policies to prevent and contain COVID-19 because of the country's large population and limited medical resources (11). The “Dynamic zero-COVID” policy was instituted, meaning that once an individual became infected, he or she was isolated in a designated location, and close contacts were sought out immediately and isolated for at least 14 days, regardless of whether their nucleic acid detection results were negative (12). The extended period of isolation inevitably led to little social interaction, an inability to work, heavy financial pressures and even bankruptcy (13), which might bring about a psychological burden and lead to symptoms of anxiety and depression (14). Moreover, people were easily infected because of the highly contagious nature of the newly evolved virus (15). Therefore, the risk of infection was high, and new outbreaks of COVID-19 continue to make the risk a stressor for the public in China. Hence, it is vital to study the mental health change trend during the repeated outbreak of COVID-19 from the aspects of stress, anxiety and depression.

**Figure 1:**
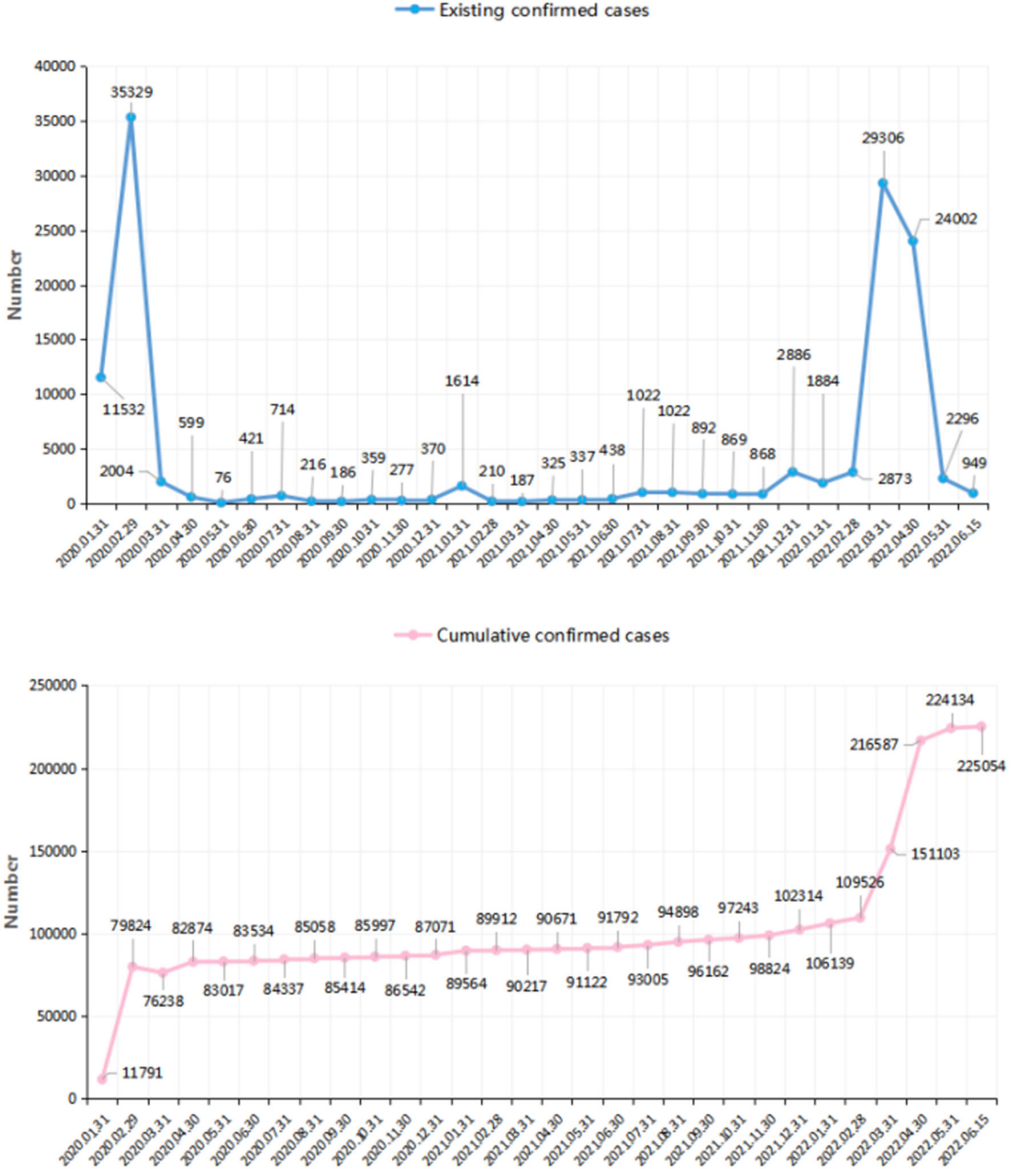
The confirmed cases of patients infected of COVID-19 in China over time (the number came from the data released by the National Health Commission of the People's Republic of China. http://www.nhc.gov.cn/xcs/yqtb/list_gzbd.shtml).

Although it has been more than 2 years since the pandemic was declared, local COVID-19 outbreaks in China have continued, including one in Nanjing and the more recent and severe outbreak in Shanghai (16). Figure 1 shows the trajectory and number of infected persons in China. With the number of infected persons increasing at different times, little is known about the mental health of the public since the initial outbreak. Although studies have been conducted using longitudinal methods to investigate the impact of COVID-19, these studies have focused only on short-term influences. One report revealed a statistically but not clinically significant reduction in psychological impact 4 weeks after the outbreak (17). Another study found no increase in the prevalence of anxiety and depression 2 months after the COVID-19 outbreak compared with pre-outbreak data (18). Meanwhile, Li et al. found that compared with the level of stress measured during the initial outbreak, acute stress declined 2 months later, while the rates of depressive and anxious symptoms increased (19). Few studies have focused on the long-term impact of COVID-19 on psychological status, with the longest follow-ups being 6 months to 1 year following the outbreak; the sample in these studies was heterogeneous (20, 21), which may have decreased the reliability of the results.

It has been reported that mental health problems, such as anxiety and depression in persons who experience major disasters (22), might persist for a long time. However, only a small number of studies have examined the psychological distress of the general public in Hubei Province (23–25), which was the first severely impacted area, and these studies were all cross-sectional surveys without follow-up data. Considering the recent conditions, especially the outbreak in Shanghai and the strict “Dynamic zero-COVID” policy, residents from Hubei Province who experienced the initial outbreak of COVID-19 still face the danger of coming into contact with infected persons. We hypothesized that the mental health of these local residents might show a distinct trend over time. Although many individuals may not experience the next outbreaks in other cities, the news and concerns about being infected by others coming from outbreak areas and the change of life brought by the Dynamic zero-COVID policy place unrelenting pressure on them. Hence, longitudinal analyses of this special group's adaptation to uncertain conditions during the pandemic are important, as the processes of cultivating an individual's resilience might change dynamically over time (26).

Therefore, we designed this longitudinal study to understand the changes in mental health from the aspects of stress, anxiety and depression among Hubei Province residents at the initial outbreak (T1), 1 month after the outbreak (T2), 18 months after [T3, the phase of another relatively large-scale outbreak in Nanjing that resulted in 1,272 newly infected individuals (27)], and 26 months after (T4, the phase of the largest-scale outbreak in China to date in Shanghai) among the same sample of Chinese residents who had experienced the initial outbreak in Hubei Province. The objective of this study was to describe the change in mental health over time among the individuals who came from the area most severely impacted by the pandemic and to explore whether the rebound of the pandemic in other cities might have an impact on them. Moreover, we also expected to discover the long-term influencing factors associated with their mental health. Only by understanding the characteristics of psychological changes and the related influencing factors can we make further plans for the subsequent management of COVID-19 and face challenges more confidently.

## 2. Materials and methods

### 2.1. Participants

This prospective study was initiated when the WHO announced COVID-19 as a Public Health Emergency of International Concern (PHEIC) on January 30, 2020, and was continued until February 19, 2020, representing the most severe period of the pandemic (T1) when the number of people infected with COVID-19 had reached 72,458 (28). The follow-up surveys were conducted from March 1–15, 2020 (T2), July 30 to August 13, 2021 (T3), and April 3–17, 2022 (T4).

Only adult (aged≥18 years) residents of Chinese nationality who had lived in Hubei Province since the COVID-19 outbreak were recruited; those who had left Hubei since the outbreak started were excluded. The first survey was conducted using convenience and snowball sampling. We sent the first batch of questionnaires to several community WeChat groups whose members consisted mostly of residents living in Hubei and encouraged everyone to forward the questionnaire link to as many groups as possible. At the end of the questionnaires, there was an invitation to participate in the follow-up surveys. If participants responded positively, they were asked to provide their WeChat account information. If they declined, no follow-up surveys were sent to them. The links for the second, third and fourth surveys were sent to the participants through their WeChat accounts to collect longitudinal data. When sending the second link, we referred to the date when the participant answered the questionnaire for the first time to ensure that the time interval was close to 1 month. Considering the occupational particularity, we added an item asking whether they were medical staff; if they answered yes, their data were excluded.

**Figure 2:**
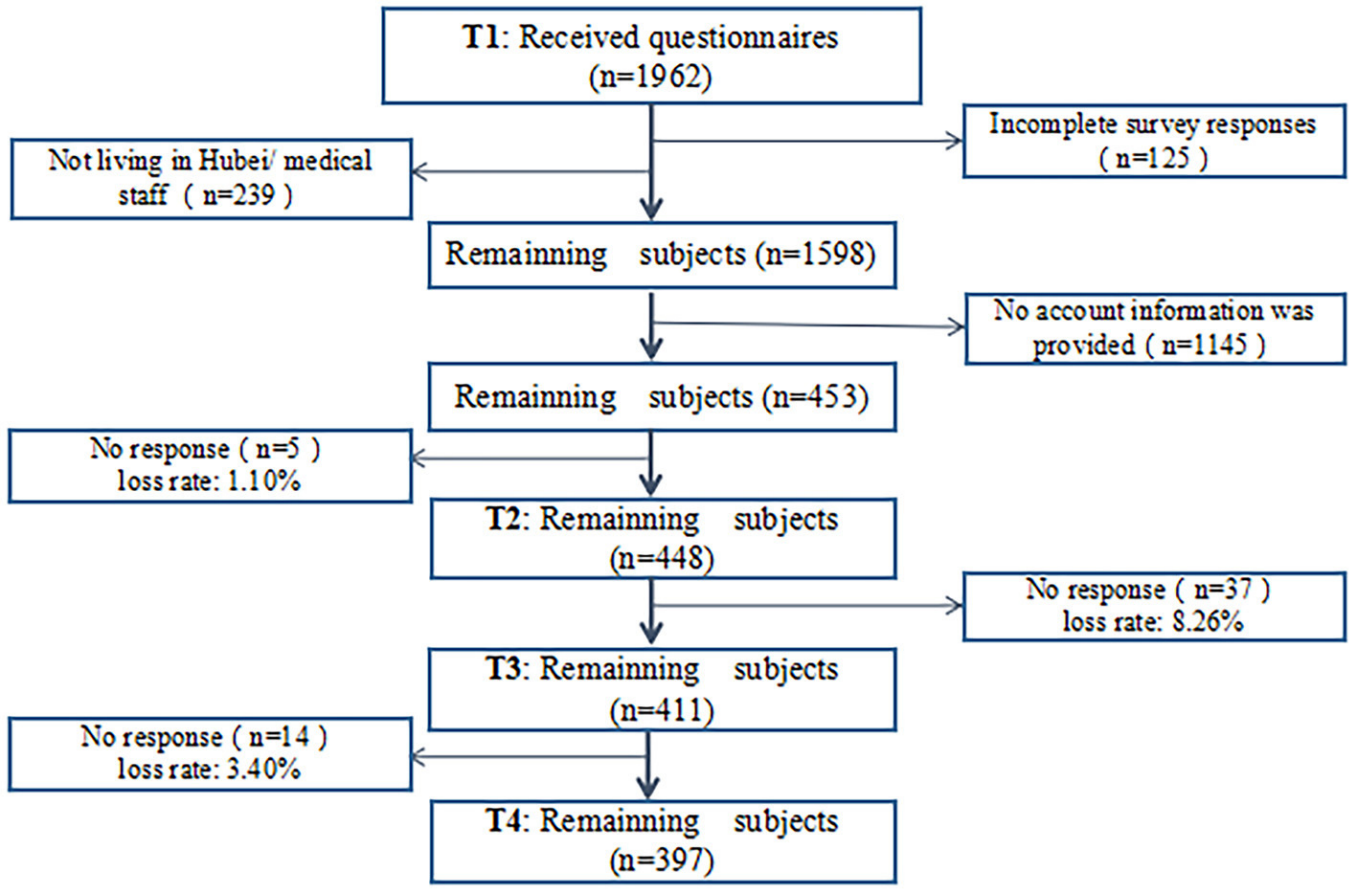
The concrete flow of subject loss.

When planning the sample size, we referred to M. Kendall's sample size estimation method, which states that the sample size should be 5–10 times the maximum number of questionnaire items (29). There were 26 questionnaire items in total; thus, the sample for this study should include 130–260 people. Considering the possibility of invalid questionnaires, the sample size was expanded by 20%. Finally, the sample size of this study was estimated to be 156–312 people. Because this is a cohort study, the research result was considered acceptable if the final sample size reached the above range. A total of 1,962 participants were recruited for the first survey. The concrete flow of subject loss is shown in Figure 2. Among the participants, only 453 provided their WeChat account information. As a result, the sample size at T1 was only 453. After we sent the second, third, and fourth surveys to these subjects, the number of subjects who returned their questionnaires was 448 at T2, 411 at T3, and 397 at T4, respectively. There was no difference in demographic characteristics among subjects at any of the four times.

Consent to participate in our survey was obtained *via* an online informed consent form provided on the first page of the questionnaire. If participants were willing to take part in the study, they chose the agree button to proceed to the questionnaire. If they selected disagree, they were returned to the home screen. All subjects were recruited voluntarily. The research design received institutional review board (IRB) approval from The Fourth People's Hospital of Chengdu.

### 2.2. Measures

#### 2.2.1. Questionnaire on demographic characteristics and experiences related to the pandemic

The questionnaire on demographic characteristics included questions on gender, age, marital status, highest education, employment status, and history of psychosis. Experiences related to the pandemic referred to whether participants had been infected with COVID-19, whether their family members had been infected with COVID-19, and whether they had been quarantined during the pandemic.

#### 2.2.2. Self-perceived health status

To determine the subjects' self-perceived health status, we added a single item based on a 5-point evaluation: 1 point indicated a very good physical condition; 2 points indicated a good physical condition; 3 points indicated an average physical condition; 4 points indicated a poor physical condition, and 5 points indicated a very poor physical condition.

#### 2.2.3. Perceived Stress Scale (PSS)

The PSS (30) is a self-assessment scale developed by Cohen et al. to assess the degree of stress an individual has felt in the past month. The PSS-10 used in this study included 10 items, including 6 items with negative descriptions (items 1, 2, 3, 6, 9, and 10) and 4 items with positive descriptions (items 4, 5, 7, and 8). Each item is scored on a 5-point scale ranging from 0 to 4. The total score is the sum of the scores for all items. The higher the score is, the greater the stress the individual has experienced. The Chinese version of the scale has proven to have good reliability and validity, with a Cronbach's alpha value of 0.83 (31).

#### 2.2.4. Generalized Anxiety Scale (GAD-7)

The GAD-7 (32) consists of seven items, each scored from 0 to 3 points; the total score ranges from 0 to 21 points, where 0 to 4 points indicates no anxiety, 5–9 points indicates mild anxiety, 10–14 points indicates moderate anxiety, and 15–21 points indicates severe anxiety. The scale has been used in China for many years and has proven to have good reliability and validity in determining the severity of anxiety. The Cronbach's alpha value of this scale is 0.898 (32).

#### 2.2.5. 9-item Patient Health Questionnaire (PHQ-9)

The PHQ-9 (33) consists of 9 items, each scored 0–3 points; the total score ranges from 0 to 27 points, where 0–4 points indicates no depression, 5–9 points indicates mild depression, 10–14 points indicates moderate depression, 15–19 points indicates moderately severe depression, and 20–27 points indicates severe depression. The scale has been used in China for many years and has proven to have good reliability and validity in determining the severity of depression. The Cronbach's alpha value of this scale is 0.86 (34).

### 2.3. Statistical analysis

We summed each total score according to the calculation rules of each scale. The mean scores are presented as the mean ± SD. To describe the longitudinal change in the mean PSS, GAD-7, and PHQ-9 scores among different time points, repeated-measures ANOVA was used. If the results of Mauchly's test led to the rejection of sphericity, Greenhouse–Geisser correction was used to adjust the degrees of freedom for the averaged tests of significance. The effect sizes were indicated by the partial eta squared value. Additionally, *post-hoc* tests for paired comparisons with Bonferroni correction were conducted to determine which mean scale scores differed significantly from others at different time points. To detect the disparity among the four time points in the rates of different levels of anxiety and depression, we used the method of crosstabs (2^*^4). Because the expected count for each tab was over 5, the Pearson chi-square was used to test whether there was a significant difference among the four time points. If the *P*-value of the chi-square was <0.05, we considered it to be a significant difference, and then further paired comparisons of the rates were conducted. The α level with Bonferroni correction was used to determine the significance. As a result, a *P*-value of <0.0125 was considered statistically significant. To build a model of the influencing factors of stress, anxiety, and depression, considering that the total scores of the above three scales were all close to a normal distribution (please see the results of normality test in Supplementary file), we used multiple linear regression with the enter method and included all independent variables to obtain comprehensive results. A *P*-value of <0.05 was considered statistically significant. The statistical software used for all analyses was SPSS, version 20.0 (IBM-SPSS Inc., Armonk, NY, USA).

## 3. Results

### 3.1. Demographic characteristics and experiences related to the pandemic at different time points

The survey included 397 participants who completed all four follow-ups, including 83 men (20.9%) and 314 women (79.1%). The average age at T1 was 44.26 ±11.38 years, ranging from 19 to 78 years. At T1, 5 participants reported a history of psychosis, including 2 with depression, 1 with bipolar disorder, and 2 with anxiety disorder. At T2, the number increased to 9, which included another 4 subjects newly diagnosed with psychosis, including 1 with depression and 3 with anxiety disorder. At T3, another 7 subjects reported a history of psychosis, including 2 with depression, 1 with drug-induced mental disorder, and 4 with anxiety disorders. At T4, the number of subjects diagnosed with psychosis was 24, with 8 newly increased subjects, including 6 with depression and 2 with anxiety disorder (all diagnoses were made by a psychiatrist). The change in the remaining demographic characteristics and the experiences related to the pandemic at different time points are shown in Table 1.

**Table 1:** Demographic and related pandemic information distribution of participants at different time points (*n* = 397).

**Variables and assignment**	**T1, *N* (%)**	**T2, *N* (%)**	**T3, *N* (%)**	**T4, *N* (%)**
**Gender**				
Man (1)	83 (20.9)	–	–	–
Woman (2)	314 (79.1)			
**Marriage**				
Unmarried (1)	44 (11.1)	44 (11.1)	44 (11.1)	42 (10.6)
Married (2)	331 (83.4)	331 (83.4)	331 (83.4)	333 (83.9)
Divorced (3)	13 (3.3)	13 (3.3)	13 (3.3)	13 (3.3)
Widowed (4)	9 (2.3)	9 (2.3)	9 (2.3)	9 (2.3)
**Highest education**				
Primary school (1)	0	0	0	0
Junior middle school (2)	16 (4.0)	16 (4.0)	16 (4.0)	16 (4.0)
Secondary specialized school (3)	12 (3.0)	12 (3.0)	12 (3.0)	12 (3.0)
High school (4)	19 (4.8)	19 (4.8)	19 (4.8)	19 (4.8)
Junior college (5)	45 (11.3)	45 (11.3)	45 (11.3)	45 (11.3)
Undergraduate (6)	259 (65.2)	259 (65.2)	259 (65.2)	259 (65.2)
Graduate (7)	46 (11.6)	46 (11.6)	46 (11.6)	46 (11.6)
**Employment status**				
Employed (1)	349 (87.9)	349 (87.9)	348 (87.7)	344 (86.6)
Not working (2)	48 (12.1)	48 (12.1)	49 (12.3)	53 (13.4)
**History of psychosis**				
Yes (1)	5 (1.3)	9 (2.3)	16 (4.0)	24 (6.0)
No (2)	392 (98.7)	388 (97.7)	381 (96.0)	373 (94.0)
**Self-perceived health conditions**				
Very good (1)	48 (12.1)	48 (12.1)	46 (11.6)	46 (11.6)
Good (2)	201 (50.6)	201 (50.6)	201 (50.6)	195 (49.1)
Average (3)	140 (35.3)	140 (35.3)	142 (35.8)	146 (36.8)
Poor (4)	8 (2.0)	8 (2.0)	8 (2.0)	10 (2.5)
Very poor (5)	0	0	0	0
**COVID-19 infection-self**				
Yes (1)	6 (1.5)	8 (2.0)	9 (2.3)	11 (2.8)
No (2)	391 (98.5)	389 (98.0)	390 (97.7)	386 (97.2)
**Isolation or not**				
Yes (1)	133 (33.5)	137 (34.5)	163 (41.1)	188 (47.4)
No (2)	264 (66.5)	260 (65.5)	234 (58.9)	209 (52.6)
**COVID-19 infection-family member**				
Yes (1)	35 (8.8)	38 (9.6)	40 (10.1)	52 (13.1)
No (2)	362 (91.2)	359 (90.4)	357 (89.9)	345 (86.9)

### 3.2. Results of the mean scores of the PSS, GAD-7, and PHQ-9 over time

**Figure 3:**
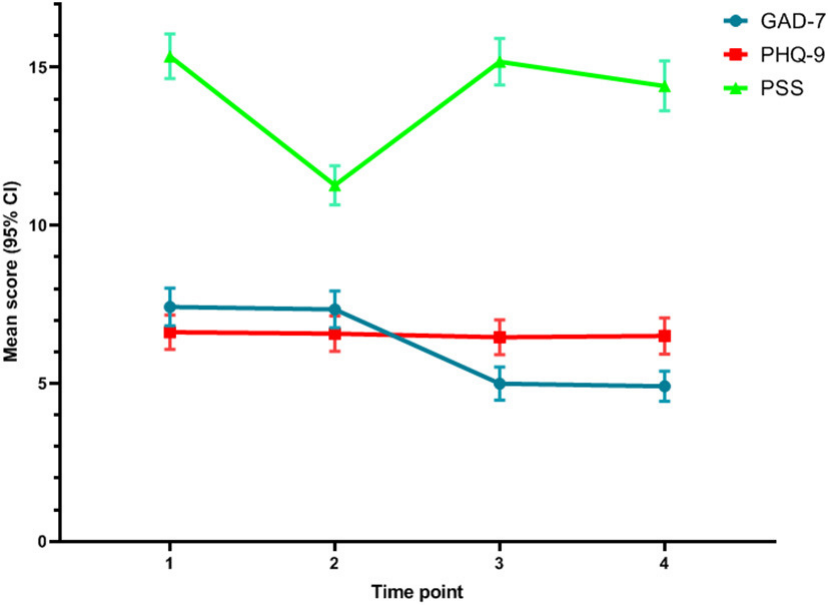
The disparity among the four time-points on the mean scores of PSS, GAD-7, and PHQ-9. The results of paired comparisons on PSS: The score at T1 was significantly higher than that at T2 (*P* < 0.001). The scores at T1 and T3 were not significantly different (*p* = 0.243). The mean score at T1 was significantly higher than that at T4 (*P* < 0.001). The score at T3 was significantly higher than that at T2 (*P* < 0.001). The score at T4 was significantly higher than that at T2 (*P* < 0.001). The score at T3 was significantly higher than that at T4 (*P* < 0.001). The results of paired comparisons on GAD-7: The scores at T1 and T2 were not significantly different (*p* = 0.310). The score at T1 was significantly higher than that at T3 (*P* < 0.001). The score at T1 was significantly higher than that at T4 (*P* < 0.001). The score at T2 was significantly higher than that at T3 (*P* < 0.001). The score at T2 was significantly higher than that at T4 (*P* < 0.001). The scores t T3 and T4 were not significantly different (*P* = 1.000). The results of paired comparisons on PHQ-9: There were no significant differences between any scores on any stage (*P* > 0.05).

The mean scores of the PSS, GAD-7, and PHQ-9 are shown in Table 2. Repeated measures ANOVA demonstrated that there were significant differences in the PSS and GAD-7 scores among the four time points [*F*_(1.96, 777.64)_ = 183.98, *p* < 0.001; *F*_(1.49, 590.26)_ = 242.00, *p* < 0.001]. There were no significant differences in the PHQ-9 scores among the four time points [*F*_(1.88, 743.10)_ = 1.256, *p* = 0.284]. Through the pairwise comparisons, the order for PSS scores at different time points was: T1/T3> T4 > T2. The order for GAD-7 scores was: T1/T2 > T3/T4. The concrete results of pairwise comparisons are listed in the footnote of Figure 3 to demonstrate which means of the variables differed from others at various time points.

**Table 2:** Repeated-measures ANOVA: the disparity among the four time-points on the mean scores of PSS, GAD-7, and PHQ-9 (*n* = 397).

	**T1 (Mean ±SD)**	**T2 (Mean ±SD)**	**T3 (Mean ±SD)**	**T4 (Mean ±SD)**	***F***	***p***	***η^2^***
PSS	15.35 ± 7.14	11.27 ± 6.27	15.17 ± 7.46	14.41 ± 7.99	183.98	< 0.001	0.317
GAD-7	7.42 ± 6.03	7.35 ± 5.88	5.00 ± 5.30	4.91 ± 4.81	242.00	< 0.001	0.379
PHQ-9	6.62 ± 5.52	6.58 ± 5.65	6.46 ± 5.58	6.50 ± 5.77	1.256	0.284	0.003

Figure 3 illustrates the change trend in the mean scores of the PSS, GAD-7 and PHQ-9. The figure shows that the mean PSS score was the highest at T1, then declined sharply at T2, showed an obvious rebound at T3, and finally decreased slightly at T4. Regarding the mean GAD-7 score, from T1 to T2, the line remained flat, while it showed an apparent decline at T3 and remained flat at T4. For the mean PHQ-9 score, the line remained flat without significant change.

### 3.3. The rates of different degrees of anxiety and depression over time

**Figure 4:**
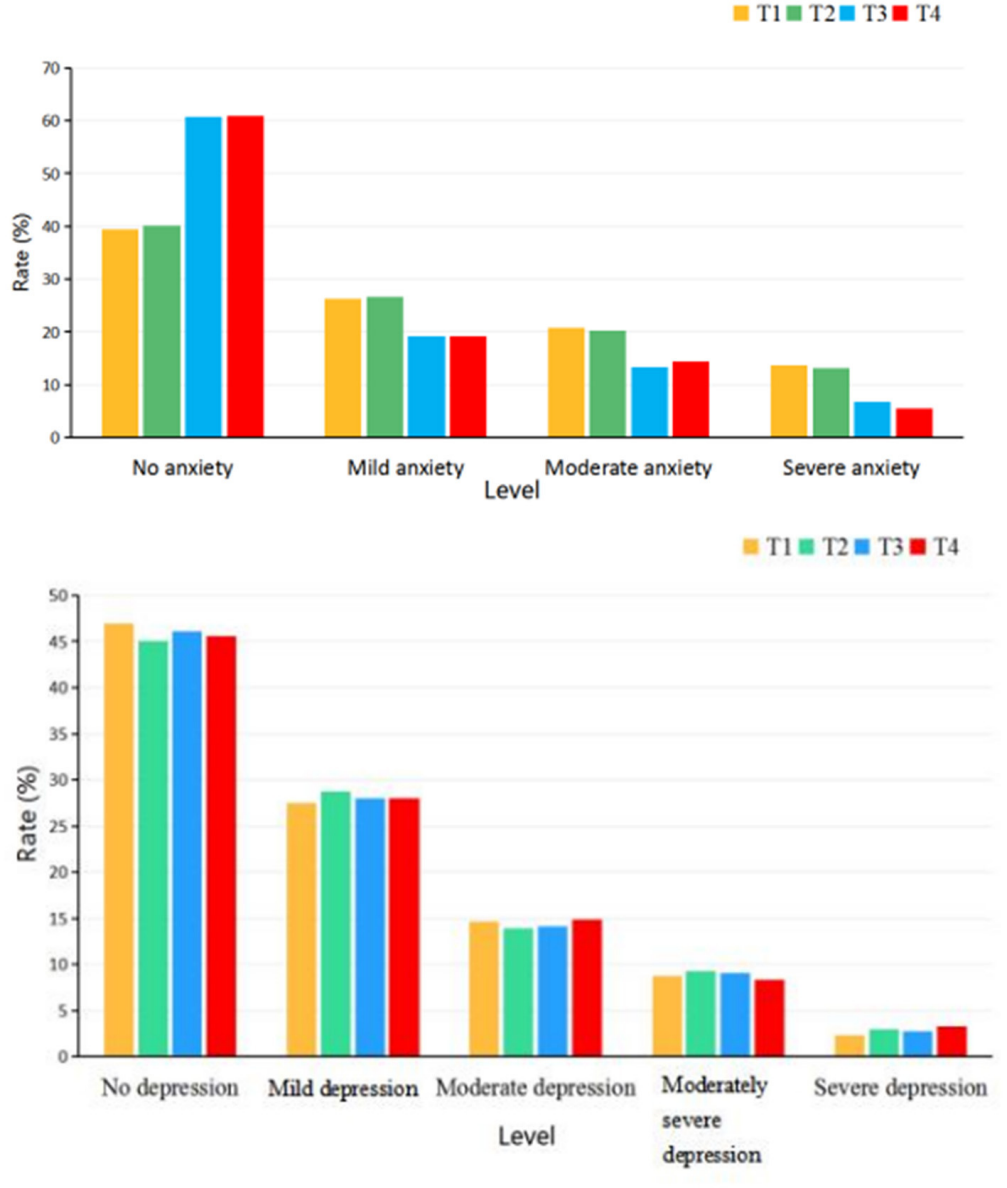
The rates of different degrees of anxiety and depression symptoms among different time points. The paired comparisons of the rates of no anxiety: T1 vs.T2 showed no significant difference (χ^2^= 0.021, df = 1, *p* = 0.942); T3 vs.T4 showed no significant difference (χ^2^= 0.005, df = 1, *p* = 1.000); T3 is higher than T1 and T2 significantly (χ^2^= 37.547; 33.876, df = 1, *p* < 0.0125); T4 is higher than T1 and T2 significantly (χ^2^= 36.399; 34.709, df = 1, *p* <0.0125). The paired comparisons of the rates of mild anxiety: T1 vs.T2 showed no significant difference (χ^2^= 0.026, df = 1, *p* = 0.936); T3/T4 showed no significant difference compared with T1 and T2 (χ^2^= 5.632; 6.416, df = 1, *p* = 0.022; 0.014). The paired comparisons of the rates of moderate anxiety: T1 vs.T2 showed no significant difference (χ^2^= 0.031, df = 1, *p* = 0.930); T3 vs.T4 showed no significant difference (χ^2^= 0.169, df = 1, *p* = 0.758); T3 is lower than T1 significantly (χ^2^= 7.506, df = 1, *p* = 0.008); T3 vs.T2 showed no significant difference (χ^2^= 6.584, df = 1, *p* = 0.013); T4 showed no significant difference compared with T1 and T2 (χ^2^= 4.666; 5.651, df = 1, *p* = 0.039; 0.025). The paired comparisons of the rates of severe anxiety: T1 vs.T2 showed no significant difference (χ^2^= 0.044, df = 1, *p* = 0.917); T3 vs.T4 showed no significant difference (χ^2^= 0.544, df = 1, *p* = 0.556); T3 is lower than T1 and T2 significantly (χ^2^= 10.022; 8.786, df = 1, *p* = 0.002; 0.004); T4 is lower than T1 and T2 significantly (χ^2^= 14.900; 13.412, df = 1, *p* < 0.0125).

Table 3 shows the rates of different degrees of anxiety and depression among the four time points. The rates of no anxiety, mild anxiety, moderate anxiety, and severe anxiety among the four time points were all significantly different (no anxiety: 39.5% at baseline vs. 40.1% at month 1 vs. 60.7% at month 18 vs. 61.0% at month 26, χ^2^ = 70.277, df = 1, *p* < 0.001; mild anxiety: 26.3% at baseline vs. 26.7% at month 1 vs. 19.1% at month 18 vs. 19.1% at month 26, χ^2^ = 12.065, df = 1, *p* = 0.007; moderate anxiety: 20.7% at baseline vs. 20.2% at month 1 vs. 13.4% at month 18 vs. 14.4% at month 26, χ^2^ = 12.173, df = 1, *p* = 0.007; severe anxiety: 13.6% at baseline vs. 13.1% at month 1 vs. 6.8% at month 18 vs. 5.5% at month 26, χ^2^ = 23.643, df = 1, *p* < 0.001). However, the rates of different degrees of depression showed no significant difference among the four time points (*p* > 0.05). To understand the differences between any two rates, we also used paired comparisons, and the detailed comparison results are listed in the footnote of Figure 4.

**Table 3:** Chi-squared test: the rates of different degrees of anxiety and depression symptoms among different time points.

	**T1, % (*n*)**	**T2, % (*n*)**	**T3, % (*n*)**	**T4, % (*n*)**	***χ^2^***	***p***
**GAD-7**						
No anxiety	39.5 (157)	40.1(159)	60.7 (241)	61.0 (242)	70.277	< 0.001
Mild anxiety	26.3 (104)	26.7 (106)	19.1(76)	19.1 (76)	12.065	0.007
Moderate anxiety	20.7 (82)	20.2 (80)	13.4 (53)	14.4 (57)	12.173	0.007
Severe anxiety	13.6 (54)	13.1 (52)	6.8 (27)	5.5 (22)	23.643	< 0.001
**PHQ-9**						
No depression	46.9 (186)	45.1 (179)	46.1 (183)	45.6 (181)	0.271	0.965
Mild depression	27.5 (109)	28.7 (114)	28.0 (111)	28.0 (111)	0.159	0.984
Moderate depression	14.6 (58)	13.9 (55)	14.1 (56)	14.9 (59)	0.205	0.977
Moderately severe depression	8.8 (35)	9.3 (37)	9.1 (36)	8.3 (33)	0.272	0.965
Severe depression	2.3 (9)	3.0 (12)	2.8 (11)	3.3 (13)	0.800	0.849

### 3.4. Multiple linear regression analysis of influencing factors of the PSS, GAD-7, and PHQ-9 total scores at T4

To explore the long-term influencing factors on the mental health of the subjects, we only considered the PSS, GAD-7, and PHQ-9 scores at T4 as the dependent variables. The reason we skipped the process of analyzing influencing factors at other time points is that previous studies have investigated these factors at similar times (19–21). To avoid repeated results, we only analyzed the longest time point as we have known to discover whether some new factors could be screened out. We also wanted to know whether previous pandemic-related experiences still have a significant impact on mental health after a long time. In the regression models, we selected demographic characteristics and pandemic experiences as the independent variables. Table 1 shows the assignments of these categorical variables entered into the models, in which numbers in the brackets after the variables' names represent the specific values.

Table 4 shows the results of the influencing factors of the PSS score. The results suggest that the main factors that influenced the subjects' feelings of stress were age, history of psychosis at T1/T4, self-perceived health condition, infection of family members by COVID-19 at T1/T4, and quarantine status at T1/T2/T3/T4 (*P* < 0.05). Table 5 shows the results of the influencing factors of the GAD-7 score. The main factors affecting the subjects' anxiety were infection of family members by COVID-19 at T1, quarantine status at T1, COVID-19 infection at T2, and history of psychosis at T4 (*P* < 0.05). Table 6 shows the results of the influencing factors of the PHQ-9. The main factors affecting the subjects' depression were age, self-perceived health condition, infection of family members by COVID-19 at T1/T3, quarantine status at T1/T3, history of psychosis at T4, and employment status at T4 (*P* < 0.05). The *F*-values (25, 371) in the regression equation were 12.461, 21.405, and 16.105 (*P* < 0.001) for PSS, GAD-7, and PHQ-9 scores, respectively, which demonstrates the statistical significance of the regression equations. The coefficients of determination (expressed as *R*^2^) were 0.456, 0.591, and 0.520 for regression models of PSS, GAD-7, and PHQ-9 scores. The screened influencing factors can effectively explain 45.6, 59.1, and 52.0% of the variance in the feelings of stress, anxiety, and depression of the subjects, respectively.

**Table 4:** Multiple linear regression analysis of influencing factors of PSS.

**Variable**	**Regression coefficients**	**Standard error of regression coefficient**	**Standardized regression coefficient**	***t***	***p***	**95% CI**
Constant	100.411	24.359		4.122	< 0.001	(52.511, 148.311)
Age	−0.088	0.039	−0.125	−2.273	0.024	(−0.164, −0.012)
History of psychosis at T1	−10.947	2.929	−0.153	−3.737	< 0.001	(−16.707, −5.186)
History of psychosis at T4	−5.686	2.426	−0.100	−2.343	0.020	(−10.456, −0.915)
Self-perceived health conditions	2.314	0.469	0.201	4.936	< 0.001	(1.392, 3.235)
COVID-19 infection-family member at T1	−5.634	1.135	−0.200	−4.963	< 0.001	(−7.867, −3.402)
COVID-19 infection-family member at T4	−6.641	1.800	−0.142	−3.690	< 0.001	(−10.180, −3.102)
Isolation or not at T1	−5.217	0.737	−0.309	−7.080	< 0.001	(−6.666, −3.768)
Isolation or not at T2	−8.060	3.595	−0.101	−2.242	0.026	(−15.128, −0.992)
Isolation or not at T3	−5.984	1.438	−0.185	−4.160	< 0.001	(−8.813, −3.156)
Isolation or not at T4	−5.049	1.330	−0.154	−3.796	< 0.001	(−7.664, −2.434)

F_(25, 371)_ = 12.461 (p < 0.001), R = 0.676, R^2^ = 0.456.

**Table 5:** Multiple linear regression analysis of influencing factors of GAD-7.

**Variable**	**Regression coefficients**	**Standard error of regression coefficient**	**Standardized regression coefficient**	***t***	***p***	**95% CI**
Constant	67.790	12.734		5.324	< 0.001	(42.751, 92.830)
COVID-19 infection-family member at T1	−5.052	0.594	−0.298	−8.513	< 0.001	(−6.219, −3.885)
Isolation or not at T1	−5.611	0.385	−0.551	−14.567	< 0.001	(−6.368, −4.853)
COVID-19 infection-self at T2	−4.943	2.388	−0.073	−2.070	0.039	(−9.640, −0.247)
History of psychosis at T4	−5.419	1.268	−0.158	−4.273	< 0.001	(−7.913, −2.925)

F_(25, 371)_ = 21.405 (p < 0.001), R = 0.768, R^2^ = 0.591.

**Table 6:** Multiple linear regression analysis of influencing factors of PHQ-9.

**Variable**	**Regression coefficients**	**Standard error of regression coefficient**	**Standardized regression coefficient**	***t***	***p***	**95% CI**
Constant	62.342	16.510		3.776	< 0.001	(29.876, 94.807)
Age	−0.064	0.026	−0.126	−2.443	0.015	(−0.116, −0.012)
Self-perceived health conditions	0.972	0.318	0.117	3.061	0.002	(0.348, 1.597)
COVID-19 infection-family member at T1	−4.840	0.770	−0.238	−6.290	< 0.001	(−6.354, −3.327)
COVID-19 infection-family member at T3	−7.296	3.359	−0.090	−2.172	0.030	(−13.901, −0.691)
Isolation or not at T1	−4.869	0.499	−0.399	−9.750	< 0.001	(−5.851, −3.887)
Isolation or not at T3	−2.209	0.975	−0.095	−2.266	0.024	(−4.127, −0.292)
History of psychosis at T4	−7.456	1.644	−0.182	−4.534	< 0.001	(−10.690,−4.222)
Employment status at T4	2.924	0.999	0.173	2.928	0.004	(0.960, 4.888)

F_(25, 371)_ = 16.105 (p < 0.001), R = 0.721, R^2^ = 0.520.

## 4. Discussion

This study aimed to understand the change trend in mental health over time and the long-term influencing factors of the residents who experienced the initial outbreak of the COVID-19 pandemic. A total of 397 participants completed all follow-ups, and the results showed that mental health changes, including stress, anxiety, and depression, differed from each other and that the depression level showed minor changes during the pandemic. Simultaneously, the long-term predictors of stress, anxiety, and depression included various demographic characteristics and experiences related to the pandemic.

Notably, the mean PSS score decreased dramatically 1 month after the initial outbreak of COVID-19 compared with the score at T1, demonstrating that the residents living in Hubei gradually adapted to the stress caused by the pandemic in the short term. The findings are similar to those reported by Li et al., which revealed that the prevalence of probable acute stress decreased among college students in China when the pandemic was under control 6 weeks after the outbreak (19). Wang et al. found that there were no significant temporal changes in the levels of stress between the initial phase and 4 weeks later during the COVID-19 pandemic in China (17). The potential explanations for the varied results could be the differences in scales, measures, and the time when the surveys were conducted, either immediately after the outbreak or later during the pandemic (35). However, to our knowledge, this is the first study that analyzed the long-term stress of a distinct group during the pandemic. We found that 1.5 years later, a new outbreak could still induce an acute feeling of stress, with the PSS score at T3 showing no significant difference compared with the score at T1, even though the pandemic mainly affected other provinces. When the outbreak with the highest number of infected individuals occurred 26 months later in another province, it still provoked a stress reaction, which could be verified by the rebound of the PSS score at T4, and the reaction was relatively smaller than that at T3. The reason for this might be that repeated outbreaks of the pandemic have made people languid. Although the large infection numbers could still trigger their stress reaction, their energy was exhausted to some extent. However, the results remind us that the impact of the pandemic on people's stress cannot be ignored, especially among those who experienced the initial outbreak. The repeated outbreaks in other areas served as triggers, which could be explained by the flashback symptoms and the cues associated with their experience in the first outbreak, which may function as warning signals to avoid future danger (36).

We also discovered that the mean score of anxiety at baseline was similar to that 1 month later. However, anxiety at T3 showed obvious differences, manifested by a significant decrease 18 months after the outbreak, and it remained at a low level 26 months after the initial outbreak. The same trajectory could also be found in the corresponding rates of various levels of anxiety. Similar to other longitudinal studies over a short period, Wang et al. reported no significant longitudinal changes in anxiety levels 4 weeks after the outbreak among the general population in China (17), and Hyland et al. also found no significant changes in the prevalence of anxiety during the 6-week lockdown caused by COVID-19 in the Republic of Ireland (37). However, different from others' results that disclosed common anxiety remaining among different types of populations due to the long-term impact of COVID-19 (38, 39), we found that anxiety showed an obvious decrease in the long term. One reason might be the special sample in our study and the special control policy in China. Another probable explanation might be that the repeated outbreak of COVID-19 has exhausted the worries of local residents, and their symptoms have gradually changed into depression. The unique finding could also be echoed by the change trend of depression described below.

Regarding the change trend of depression, we found that in both the short term and the long term, the depression level did not change significantly. Although some studies also revealed a relatively stable level of depression (17, 37) in a short time after the outbreak of COVID-19, Yuan et al. discovered a significant improvement in the prevalence of depression 3 months after the outbreak in China (40). Other studies conducted in northern Spain or Southeast Asia demonstrated that the depressive symptoms persisted after 1.5 years of COVID-19 (41, 42). However, our findings make up the margin of longer-term follow-up after the outbreak of COVID-19, and indicate that the symptoms of depression among the residents who experienced the initial pandemic were difficult to eliminate with the background of repeated outbreaks in contrast to the trend of anxiety. A meta-analysis by Robinson et al. also showed that the reduction in depression over time during the COVID-19 pandemic was less pronounced than the reduction in anxiety (43). The trend of depression over time conforms to the finding of Du et al., who demonstrated that when people experience stress, anxious emotions occur first, and with the continuation of stress, this emotion gradually evolves into depressive symptoms (44). These results suggest that we should pay attention to the long-term mental health of residents experiencing catastrophic emergencies because a post-disaster psychological crisis can persist for a long time, and the onset can be delayed (45, 46). Although the “Dynamic zero-COVID” policy in China could control the spread of the virus to the maximum extent, the impact of the large-scale shutdown inevitably increased the burden on the economy, which induced negative emotions in the residents (47). Considering these results, we recommend that policymakers adjust policies appropriately in the future to minimize the negative impact of pandemic precautions.

Regarding the long-term influencing factors of stress, anxiety, and depression, we found that all these forms of psychological distress were associated with the subjects' quarantine status and whether their family members were infected during the initial outbreak. Many studies also reported that people who had been quarantined due to the pandemic showed poor mental health status (25, 48, 49). Additionally, we found that each isolation experience at a different time point could increase the long-term risk of stress, indicating that quarantine status is a great predictor of mental pressure, and the isolation experience at T3 could also predict depression. Due to the social attributes of human beings, all humans are at risk of psychological harm when in isolation (50). After people experienced the first isolation, which produces a negative psychological state, repeated isolation undoubtedly triggers subsequent negative emotions, including loneliness and sadness (51). Regarding the infection of family members, similar to the findings reported by Chen et al., people who worried about their family members being infected with COVID-19 had a higher prevalence of anxiety (52). Strong family and social support reduces anxiety and depression (53). When family members were infected, they faced separation from their support system, which had an adverse impact on their mental health, and family members' infection at T3 also increased individuals' depressive symptoms.

We also found that the subjects with a history of psychosis at T1 might experience more symptoms of stress, and those with a history of psychosis at T4 showed more symptoms not only of stress but also of anxiety and depression. Luo et al. also found that a history of mental illness was a risk factor for acute stress responses (54), and the outbreak of this pandemic was undoubtedly a crisis for those with a history of mental illness, which could affect their access to medical treatment and worsen their mental symptoms (5). Many of these individuals developed psychosis 1.5 years after the initial outbreak. The newly developed illness undoubtedly added to an individual's worried state of mind, which was exacerbated when outbreaks recurred. It is likely that this population was more vulnerable than the general population when facing these disease-related stressors (55). This fact reminds us that we must conduct crisis intervention services as early as possible and provide alternative medical treatment programs for this group to avoid mental health issues and increased social burden (56). During public health crises that require isolation and quarantine, such as COVID-19, psychological interventions such as cognitive behavioral therapy delivered *via* the internet could play a key role in treating these special groups (57).

Our results revealed that the poorer the condition individuals perceived themselves to be in and the younger their age was, the greater their probability of feeling stressed and depressed. The results were similar to those of Chen et al., who also reported that self-perceived health status tended to be positively associated with changes in stress and depression scores from 1 week to 1 month after the COVID-19 outbreak (58). One reasonable explanation is that the participants were not optimistic about their health, and they were more worried that their bodies could not resist the virus, which could make them more sensitive to the threat than ordinary people. As Wang et al. found, being satisfied with one's own health could be a protective factor for people's mental health during the pandemic (17). Among all the demographic factors, we found that gender, marriage, and education did not play a significant role in predicting the mental health of these subjects after a long time post-pandemic, which indirectly indicates the importance of pandemic-related experience factors. However, we found that the demographic factor of age is special, consistent with other longitudinal studies, indicating that depression was more common in younger populations (37, 51, 59). Considering the special condition of China, we believe that the reason for younger age triggering more stress might be that the older subjects in our study might have experienced the SARS pandemic, which occurred in 2003, while the younger subjects might not have. Thus, the sudden onset of the pandemic became a strong stressor for them.

Other single factors for the risk of anxiety and depression included being infected at T2 and being unemployed at T4. Xiao et al. also reported that COVID-19 infection might have long-term impacts on local residents' mental health (60). When people escaped successfully from the initial infection and were infected 1 month later, they inevitably enperienced a lingering fear, which might contribute to the relatively high scores on the anxiety scales. Many studies have indicated that the loss of important resources, such as employment and income, might cause chronic mental health problems (61–63); thus, unemployment status could be a long-term predictor of depression.

### 4.1. Limitations

(1) The online survey method we used might lead to non-response bias or reporting/selection bias, which could be reflected by the phenomenon that females predominated in this survey, and not all independent variables such as the gender were found to contribute to the depression model; (2) The method of Bonferroni correction used in this study might be too conservative, and it is prone to have type II errors; (3) The use of self-reported rating scales limits the diagnosis of anxiety and depression, and the scores of the scales could only suggest probable anxiety and depression. If a diagnostic interview was used, some subjects would not have met the criteria for diagnosis. However, these scales have proven to have good sensitivity and specificity, and they have been effectively used in clinical studies. Because of the voluntary nature of participation, only one-quarter of the participants in the first survey agreed to take part in the follow-up surveys, resulting in a small sample size, which might prevent the conclusions from being generalized to the larger population.

### 4.2. Conclusions

This long-term longitudinal survey revealed that the symptoms of stress among residents who experienced the initial outbreak of COVID-19 decreased in the short term, while the symptoms of anxiety and depression did not change significantly. In the long term, repeated outbreaks in other areas also impacted this distinct group, with a return of stress, a decline in anxiety, and no change in depression. The long-term influencing factors differ for stress, anxiety, and depression, but all three are influenced by a history of psychosis at T4, quarantine status and whether their family members were infected during the initial phase of the pandemic.

## Data availability statement

The raw data supporting the conclusions of this article will be made available by the authors, without undue reservation.

## Ethics statement

The studies involving human participants were reviewed and approved by Ethics Committee of the Fourth People's Hospital of Chengdu. Written informed consent for participation was not required for this study in accordance with the national legislation and the institutional requirements.

## Author contributions

ND: conception, design, and drafting of the manuscript. ND, YX, YJOY, YGL, TG, CYL, CY, YLH, FYL, LZ, MZ, LSL, and JH: conduction. ND, YGL, and TG: statistical analysis. YGL and CYL: administrative, technical, or material support. YX: critical revision of the manuscript for important intellectual content. All authors read and approved the final paper.
