# Supplementary material for: Longitudinal study of mental health changes in residents affected by an initial outbreak of COVID-19 in China
Source: Front Public Health. 2023 Jan 9;10:1019703. doi: 10.3389/fpubh.2022.1019703 (PMC9868630; doi:10.3389/fpubh.2022.1019703)
Supplement: Supplementary file 1 [file Data_Sheet_1.DOC]

**Table 1. The normality test of scale variables**

| **Variables** | **Mean** | **Median** | **Range** | **Skewness value** |
| --- | --- | --- | --- | --- |
| age | 44.3 | 45.0 | 59.0 | 0.102 |
| PSS at T1 | 15.35 | 15.0 | 33.0 | 0.217 |
| PSS at T2 | 11.27 | 11.0 | 28.0 | 0.359 |
| PSS at T3 | 15.17 | 15.0 | 34.0 | 0.186 |
| PSS at T4 | 14.41 | 15.0 | 32.0 | 0.070 |
| GAD-7 at T1 | 7.4 | 6.0 | 21.0 | 0.627 |
| GAD-7 at T2 | 7.3 | 6.0 | 21.0 | 0.591 |
| GAD-7 at T3 | 5.0 | 3.0 | 21.0 | 1.151 |
| GAD-7 at T4 | 4.9 | 3.0 | 20.0 | 1.084 |
| PHQ-9 at T1 | 6.6 | 5.0 | 23.0 | 0.861 |
| PHQ-9 at T2 | 6.58 | 6.0 | 22.0 | 0.809 |
| PHQ-9 at T3 | 6.46 | 5.0 | 22.0 | 0.827 |
| PHQ-9 at T4 | 6.50 | 5.0 | 23.0 | 0.838 |

**The questionnaires used in the study (In Chinese language)**

1. **Questionnaire on demographic characteristics and experiences related to the pandemic**

**请根据您的实际情况填写如下问题，并选择符合您情况的选项：**

1. **您的性别：**

男 女

1. **您的年龄：** 岁
2. **您的婚姻状态：**

未婚 已婚 离异 丧偶

1. **您的最高学历：**

小学 中专 初中 高中 大专 本科 研究生

1. **您的工作状态：**

无工作 有工作，请问您是否是医护工作者？ 否 是

1. **您是否有精神疾病史（需曾经被精神科医师确诊过的相应精神疾病）**：

无 有，如果有，请填写具体诊断名称：

**以下是关于您在本次新型冠状病毒肺炎疫情爆发后的相关经历，请根据您的情况进行相应选择：**

1. 截止本次调查时，您是否已经感染过新型冠状病毒：

否 是

1. 截止本次调查时，您的家人是否有人已经感染过新型冠状病毒：

否 是

1. 截止本次调查时，您是否有过被隔离的经历：

否 是

**以下是您对于自身的健康状态评估，请根据您的实际情况进行相应选择：**

**您认为您目前的身体健康状态可以用以下哪种程度形容：**

1. 非常好
2. 较好
3. 一般
4. 较差
5. 非常差
6. **Perceived Stress Scale (PSS)**

**指导语：以下问题询问你在过去一个月的一些感受和想法，对于每一个问题，请选出符合你的情况。**

1. **在过去的一个月里，你有多少时间因为发生意外的事情而感到心烦意乱？**

从未有 几乎没有 偶尔 经常 非常多

1. **在过去的一个月里，有多少时间你感到无法掌控生活中重要的事情？**

从未有 几乎没有 偶尔 经常 非常多

1. **在过去的一个月里，有多少时间你感觉到神经紧张或“快被压垮了”？**

从未有 几乎没有 偶尔 经常 非常多

1. **在过去的一个月里，有多少时间你对自己处理个人问题的能力感到有信心？**

从未有 几乎没有 偶尔 经常 非常多

1. **在过去的一个月里，有多少时间你感到事情发展和你预料的一样？**

从未有 几乎没有 偶尔 经常 非常多

1. **在过去的一个月里，有多少时间你发现自己无法应付那些你必须去做的事情？**

从未有 几乎没有 偶尔 经常 非常多

1. **在过去的一个月里，日常生活中有多少时间你能够控制自己的愤怒情绪？**

从未有 几乎没有 偶尔 经常 非常多

1. **在过去的一个月里，有多少时间你感到处理事情得心应手（事情都在你的控制之中）？**

从未有 几乎没有 偶尔 经常 非常多

1. **在过去的一个月里，有多少时间你因为一些超出自己控制能力的事情而感到愤怒？**

从未有 几乎没有 偶尔 经常 非常多

1. **在过去的一个月里，有多少时间你感到问题堆积如山，已经无法逾越？**

从未有 几乎没有 偶尔 经常 非常多

1. **Generalized Anxiety Scale (GAD-7)**

**指导语：根据过去两周的状况，请您回答是否存在下列描述的状况及频率，请看清楚问题后选择符合您的选项。**

1. **感到不安、担心及烦躁**

没有（0） 有几天（1） 一半以上时间（2） 几乎天天（3）

1. **不能停止或无法控制担心**

没有（0） 有几天（1） 一半以上时间（2） 几乎天天（3）

1. **对各种各样的事情担忧过多**

没有（0） 有几天（1） 一半以上时间（2） 几乎天天（3）

1. **很紧张，很难放松下来**

没有（0） 有几天（1） 一半以上时间（2） 几乎天天（3）

1. **非常焦躁，以至无法静坐**

没有（0） 有几天（1） 一半以上时间（2） 几乎天天（3）

1. **变得容易烦躁或易被激怒**

没有（0） 有几天（1） 一半以上时间（2） 几乎天天（3）

1. **感到好像有什么可怕的事会发生**

没有（0） 有几天（1） 一半以上时间（2） 几乎天天（3）

1. **9-item Patient Health Questionnaire (PHQ-9)**

**指导语：根据过去两周的状况，请您回答是否存在下列描述的状况及频率，请看清楚问题后选择符合您的选项。**

1. **做事时提不起劲或没有兴趣**

完全不会（0） 好几天（1） 超过一周（2） 几乎每天（3）

1. **感到心情低落、沮丧或绝望**

完全不会（0） 好几天（1） 超过一周（2） 几乎每天（3）

1. **入睡困难、睡不安稳或睡眠过多**

完全不会（0） 好几天（1） 超过一周（2） 几乎每天（3）

1. **感觉疲倦或没有活力**

完全不会（0） 好几天（1） 超过一周（2） 几乎每天（3）

1. **食欲不振或吃太多**

完全不会（0） 好几天（1） 超过一周（2） 几乎每天（3）

1. **觉得自己很糟，或觉得自己很失败，或让自己和家人失望**

完全不会（0） 好几天（1） 超过一周（2） 几乎每天（3）

1. **对事物专注有困难，例如阅读报纸或看电视时**

完全不会（0） 好几天（1） 超过一周（2） 几乎每天（3）

1. **动作或说话速度缓慢到别人已经察觉？或正好相反——烦躁或左立不安、动来动去的情况更胜于平常**

完全不会（0） 好几天（1） 超过一周（2） 几乎每天（3）

1. **有不如死掉或用某种方式伤害自己的念头**

完全不会（0） 好几天（1） 超过一周（2） 几乎每天（3）
